# A narrative review of what cohorts have taught us and how they have laid the foundation for much of our understanding of type 2 diabetes

**DOI:** 10.1007/s00125-026-06812-4

**Published:** 2026-08-26

**Authors:** Sun H. Kim, Ipsa Arora, Charles Agyemang, Ranjit M. Anjana, Bjørn Olav Åsvold, Marieke Blom, Shilpa N. Bhupathiraju, Amélie Bonnefond, Juliana C. N. Chan, Timothy M. E. Davis, Kristian Hveem, Marit E. Jørgensen, Anton Lager, Lars Lind, Olle Melander, Andrea Natali, Jackie Price, Parminder Raina, Michael Roden, Olov Rolandsson, Mary R. Rooney, Seyed Mohammad Mousavi, Andrei C. Sposito, Reimar W. Thomsen, Barbara Thorand, Rob M. Van Dam, Kavita Venkataraman, Sarah H. Wild, Ji Hee Yu, Petra J. M. Elders, Joline W. Beulens, Femke Rutters

**Affiliations:** 1 Division of Endocrinology, Gerontology and Metabolism, Department of Medicine, Stanford University School of Medicine, Stanford, CA USA; 2 Division of Endocrinology, Diabetes and Metabolism, Department of Medicine, Central Maine Medical Center, Lewiston, ME USA; 3 Department of Public and Occupational Health, Amsterdam UMC, University of Amsterdam, Amsterdam Public Health Research Institute, Amsterdam, the Netherlands; 4 Division of Endocrinology, Diabetes and Metabolism, Department of Medicine, The Johns Hopkins University School of Medicine, Baltimore, MD USA; 5 Department of Diabetology, Madras Diabetes Research Foundation & Dr. Mohan’s Diabetes Specialities Centre, ICMR Centre for Advanced Research on Diabetes, Chennai, India; 6 HUNT Center for Molecular and Clinical Epidemiology, Department of Public Health and Nursing, Norwegian University of Science and Technology (NTNU), Trondheim, Norway; 7 Department of Endocrinology, Clinic of Medicine, St Olavs Hospital, Trondheim University Hospital, Trondheim, Norway; 8 Department of General Practice, Amsterdam UMC, University of Amsterdam, Amsterdam Public Health Research Institute, Amsterdam, the Netherlands; 9 Channing Division of Network Medicine, Harvard Medical School and Brigham and Women’s Hospital, Boston, MA USA; 10 University of Lille, Inserm/CNRS UMR 1283/8199, Institut Pasteur de Lille, European Genomics Institute for Diabetes (EGID), Lille University Hospital, Lille, France; 11 Department of Medicine and Therapeutics, Li Ka Shing Institute of Health Science, Hong Kong Institute of Diabetes and Obesity, The Chinese University of Hong Kong, The Prince of Wales Hospital, Hong Kong SAR, China; 12 Medical School, University of Western Australia, Fremantle Hospital, Fremantle, WA Australia; 13 Department of Research, St Olav’s Hospital, Trondheim, Norway; 14 Steno Diabetes Center Greenland, Nuuk, Greenland; 15 Department of Global Public Health, Karolinska Institutet, Stockholm, Sweden; 16 Department of Medical Sciences, Uppsala University, Uppsala, Sweden; 17 Department of Clinical Sciences Malmö, Lund University, Malmö, Sweden; 18 Department of Internal Medicine, Skåne University Hospital, Malmö, Sweden; 19 Meta Lab, Department of Clinical and Experimental Medicine, University of Pisa, Pisa, Italy; 20 Usher Institute, School of Population Health Sciences, University of Edinburgh, Edinburgh, UK; 21 Department of Health Research Methods, Evidence, and Impact, McMaster University, Hamilton, ON Canada; 22 McMaster Institute for Research on Aging, McMaster University, Hamilton, ON Canada; 23 Labarge Centre for Mobility in Aging, McMaster University, Hamilton, ON Canada; 24 Department of Endocrinology and Diabetology, Medical Faculty and University Hospital, Heinrich Heine University, Düsseldorf, Germany; 25 German Center for Diabetes Research (DZD e.V.), Partner München-Neuherberg, Neuherberg, Germany; 26 Institute for Clinical Diabetology, German Diabetes Center, Leibniz Institute for Diabetes Research at Heinrich Heine University, Düsseldorf, Germany; 27 Department of Public Health and Clinical Medicine, Umeå University, Umeå, Sweden; 28 Department of Epidemiology and Welch Center for Prevention, Epidemiology, and Clinical Research, Johns Hopkins Bloomberg School of Public Health, Baltimore, MD USA; 29 Department of Nutrition, Harvard T. H. Chan School of Public Health, Boston, MA USA; 30 Division of Cardiology, Department of Medicine, State University of Campinas, Campinas, Brazil; 31 Department of Clinical Epidemiology, Aarhus University and Aarhus University Hospital, Aarhus, Denmark; 32 Institute of Epidemiology, Helmholtz Zentrum München, German Research Center for Environmental Health (GmbH), Neuherberg, Germany; 33 Institute for Medical Information Processing, Biometry, and Epidemiology (IBE), Faculty of Medicine, LMU Munich, Pettenkofer School of Public Health, Munich, Germany; 34 Department of Exercise and Nutrition Sciences, Milken Institute School of Public Health, The George Washington University, Washington, DC USA; 35 Department of Epidemiology, Milken Institute School of Public Health, The George Washington University, Washington, DC USA; 36 Saw Swee Hock School of Public Health, National University of Singapore, Singapore, Republic of Singapore; 37 Usher Institute for Population Health Sciences, University of Edinburgh, Edinburgh, UK; 38 Division of Endocrinology and Metabolism, Department of Internal Medicine, Korea University College of Medicine, Seoul, Republic of Korea; 39 Department of Epidemiology and Data Science, Amsterdam Public Health, Amsterdam University Medical Center, Amsterdam, the Netherlands

**Keywords:** 50 years, Cohorts, Knowledge, Review, Type 2 diabetes

## Abstract

This narrative review provides a historical perspective on how observational research on type 2 diabetes has been developed and consolidated over the last 50 years and how well-designed cohort studies will provide us with knowledge for research and practice in the future and aid guideline development. We have included data from a large number of cohorts from every continent that have been used to study the development and/or progression of type 2 diabetes, including cohorts that are general population-based, disease-based, intervention-based and registry-based. We have structured the results from the past 50 years based on the following themes: diagnosis and screening, complications, risk factors and pathophysiology. We also discuss the strengths and weaknesses of observational research when compared with other research designs. Finally, we discuss the emerging and future directions for type 2 diabetes research using cohorts, which include novel developments, such as artificial intelligence, precision health and the exposome. We conclude that cohort research has significantly advanced our understanding of type 2 diabetes and aided guideline development, and complements experimental work, such as human randomised controlled trials and animal studies. Both approaches are essential and complementary in our pursuit to provide a more comprehensive understanding of the development and progression of type 2 diabetes, and to change dogma, practice and policies for better outcomes.

**Graphical Abstract:**

**Figure:**
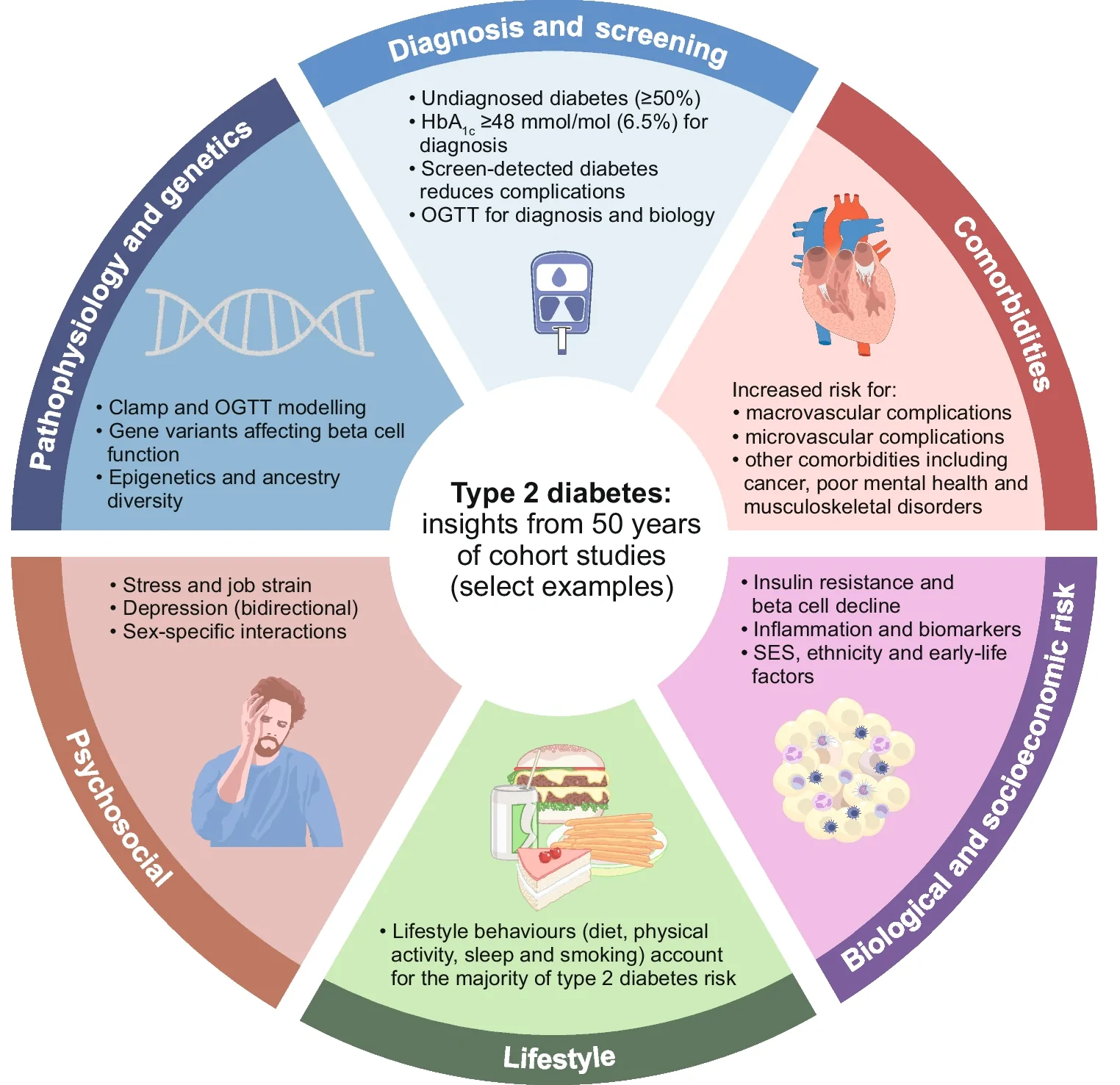

## Introduction

It was not until the late 1940s that observational research gained momentum with the implementation of several landmark prospective cohort studies, including the Framingham Heart Study (USA) [1] and the British Doctors Study [2]. In the 1950s, the British Doctors Study showed the strong association between tobacco smoking and lung cancer, underscoring the power of observational research to inform public health policy [3]. The 1980s saw a surge in cohort studies, a trend that continues today, particularly in diabetes epidemiology where new cohorts are continuously being developed. The ongoing expansion of cohort studies raises an important question: ‘What can we learn from these cohort studies?’ To address this, in this narrative review we explore what cohort studies have taught us about the development and progression of type 2 diabetes over the past 50 years and the implications for the future of the diabetes research and care landscape.

## Methods

This narrative review could not cover all type 2 diabetes cohorts. For additional resources, the REDDIE project (https://www.reddie-diabetes.eu/about) provides a comprehensive global overview of 573 type 2 diabetes cohorts across 58 countries via its online dashboard (https://jjct.quarto.pub/real-world-databases-in-diabetes/ and https://www.reddie-diabetes.eu/research/rwd-dashboard). For this narrative review, we included a convenience sample of general population-based, disease-based, intervention-based and registry-based cohorts, with a strong focus on type 2 diabetes, ensuring that there was a geographical spread with more than ten publications on type 2 diabetes from each region. In 2024, we contacted cohort investigators to obtain design papers and lessons learned and identified a representative co-author for each cohort. Tables 1 and 2 summarise the cohort characteristics and key lessons learned, stratified by European cohorts (Table 1) and cohorts from the rest of the world (Table 2). We categorised these lessons into thematic domains and contextualised them within the existing literature; however, not all lessons learned readily aligned with the domains and therefore not all are described in the main text. In addition, we discuss the strengths and limitations of observational research compared with other study methodologies.

**Table 1:** European cohorts: characteristics and key lessons learned

Cohort name Link to cohort	Representative Year started Study population size	Female participants (%)	Age at recruitment (years)	Type of cohort	Important examples
Europe (Denmark, the Netherlands, UK)					
ADDITION-Europe NA	Marit Jørgensen 2001–2006 3057	NA	40–69	Prospective population-based, started as RCT on intense T2D treatment	Early intensive treatment of screen-detected T2D showed only small, non-significant cardiovascular benefits at 5 years, although long-term follow-up may clarify effects [25]. A stepwise screening strategy (using risk questionnaires, blood glucose, HbA_1c_, etc.) can work in a general practice setting [142]. Screening did not lead to large adverse psychological effects: people did not become significantly more anxious or depressed, nor were they falsely reassured [143].
Europe					
EGIR-RISC egir-group.eu/	Andrea Natali 2002 1500 (as of 2004)	NA	30–60	Prospective participant-based	*CDKAL1* and *HHEX*/*IDE* genes are linked to decreased pancreatic beta cell function, while the *FTO* gene is associated with increased adiposity affecting insulin sensitivity [106]. Hyperglycaemia in glucose intolerance is mainly due to an intrinsic defect in beta cell glucose sensitivity rather than inadequate compensation for IR, underscoring the critical role of beta cell function in the development of glucose intolerance and T2D [107]. Fasting and post-load glucose homeostasis are largely independent, with each sustained by distinct beta cell defects. Fasting glucose disruption involves reduced beta cell sensitivity to fasting glucose, while post-load disruption affects the beta cell glucose dose–response [144].
Denmark					
ADDITION-PRO NA	Marit Jørgensen 2001–2006 2082	47	40–69	Prospective population-based	Risk groups with IFG or IGT had higher progression rates to T2D and CVD, with detailed assessments revealing early cardiometabolic complications 35]. Baseline HbA_1c_ was associated with higher aortic stiffness after 7.8 years of follow-up [145]. Higher objectively measured PAEE was strongly associated with better glucose metabolism and better insulin sensitivity. Total energy expenditure is more important than intensity alone [146].
Inter99 NA	Marit Jørgensen 1999 6004	NA	30–60	Prospective population-based, started as RCT on lifestyle intervention	Among Danish individuals aged 30–60 years, T2D prevalence is high, with a large proportion undiagnosed, underscoring the need for improved screening and intervention [16]. Inter99 has contributed data to large GWAS of BMI, waist-to-hip ratio, body fat distribution, lipids, IR and BP; early studies identified variants in *FTO*, *MC4R*, *TCF7L2*, *GCKR*, *SLC30A8* and *PPARG*; meta-analyses by GIANT, MAGIC and CARDIoGRAM identified additional associations; and gene–physical activity interactions have been reported for *FTO* [16]. In the randomised Inter99 trial (*n*~59,616 people aged 30–60 years), screening for risk factors plus repeated lifestyle counselling over 5 years did not significantly reduce the 10 year incidence of ischaemic heart disease, stroke or mortality compared with control groups [147].
LOFUS pure-portal.regsj.dk/en/organisations/lolland-falster-undersøgelsen	Marit Jørgensen 2016 20,000	NA	0–96	Prospective household-based	Diagnostic advances have lowered estimates of undiagnosed cases, yet 24% remain undiagnosed, reinforcing the importance of systematic screening and population monitoring [148]. CKD occurs frequently, is poorly recognised and overlaps strongly with metabolic diseases in this population, highlighting the need for integrated screening [149]. LOFUS provides strong data to study how socioeconomic disadvantage translates into physiological risk, e.g. higher allostatic load was associated with dysmetabolism and increased all-cause mortality [150].
DD2 dd2.dk	Reimar W. Thomsen 2010 13,500 (as of 2025)	41	17–95	Prospective patient cohort	There are three pathophysiological T2D subphenotypes (insulinopaenic, classical and hyperinsulinaemic), which manifest different patterns of clinical features and onset of complications [125]. Metabolic and lifestyle factors increase the risk of diabetic polyneuropathy in early T2D, highlighting the value of early intervention [34]. Low birthweight (<3000 g) is linked to higher cardiovascular risk and mortality in newly diagnosed T2D, mainly from stroke and CVD [151].
France					
D.E.S.I.R. NA	Amélie Bonnefond 1994 5024	50.9	47 ± 10	Prospective population-based	After T2D onset, *TCF7L2* risk variants and a FHx of diabetes jointly accelerate beta cell decline and HbA_1c_ rise, showing that genetic and familial factors together drive worsening glycaemia [152]. Metabolomics-based models accurately predict T2D, improving risk prediction by 4.5% over clinical markers and identifying metabolites associated with earlier disease onset, supporting targeted preventive interventions [153]. Women with a FHx of T2D have a higher risk of developing T2D independent of genetic risk, whereas no such association was seen in men, suggesting targeted screening for at-risk women may be warranted [154].
Germany					
GDS dzd-ev.de/en/research/multicenter-studies/german-diabetes-study-gds/	Michael Roden 2009 2188 (as of 2025)	T1D 45, T2D 37	18–69	Prospective observational, participants with new-onset diabetes and without diabetes	T2D can be classified into five subtypes (MARD, MOD, SAID, SIRD, SIDD) based on IR, adipose function and comorbidities; SIRD and MOD have high IR, liver fat and inflammation, raising risks of nephropathy and neuropathy [123]. Compared with T1D, individuals with T2D show higher liver fat, more adiposity and lower hepatic energy levels, with hepatic metabolism changes over time linked to insulin sensitivity and MASLD progression [36]. An insulin sensitivity/secretion gradient maps high liver fat, inflammation, cardiovascular risk and mortality to low sensitivity, and neuropathy/insulin needs to low secretion, supporting personalised risk assessment and treatment in T2D [124].
KORA helmholtz-munich.de/en/epi/cohort/kora	Barbara Thorand 1984/1985; 1989/1990; 1994/1995, 1999/2001 (baseline assessments) 17,602	~50	25–74	Prospective population-based	~40% of people aged 55–74 years have disturbed glucose tolerance or diabetes, with half of the diabetes cases undiagnosed; key risk factors include abdominal adiposity (men), hypertriglyceridaemia (women), hypertension and parental diabetes history, underscoring the need for targeted screening and prevention [15]. Low-grade systemic inflammation is an important risk factor for diabetes development in men aged 45–74 years [155]. In Augsburg, the prevalence of known T2D stayed stable over 17 years from 1984 to 2001. Diabetes treatment improved continuously during the study period, but poor health status and high CVD risk in individuals with diabetes stress the need for early diagnosis and better treatment for CVD prevention in people with diabetes [156].
The Netherlands					
Hoorn Studies hoornstudies.com	Femke Rutters Wave 1: 1989; Wave 2: 2006; Wave 3: 2023 (ongoing) 2484; 2807; ~3000	Wave 1 54.1; wave 2 53.5; wave 3 ~50	Wave 1 50–75; wave 2 40–65; wave 3 30–55	Prospective population-based	The association between psychosocial stress and mortality is mediated by lifestyle and chronic diseases [101]. About 5% of the total population had undetected diabetes [98]. Pre-stages of T2D, such as the metabolic syndrome, also result in higher risk of complications [157].
Diabetes Care System hoornstudies.com	Marieke Blom 1996 15,000	47.7	>18	Patient-based, T2D	The chronic care model results in greatly improved T2D care [158]. Screening models perform well for prediction of microvascular complications [159–161].
The DIAMANT cohort diabetes-diamant.nl/about-diamant/	Jetty Overbeek 2004 35,000	48	>18	Registry	Increase in the prevalence of diabetes from 1.8% in 1999 to 4.9% in 2014 in the Netherlands [162]. Individuals with T2D compared with matched participants without T2D had a 1.3 times higher risk of developing colorectal cancer [163]. Women with T2D were at an increased risk of being diagnosed with more advanced tumour stages of breast cancer (OR 1.28 [95% CI 1.13, 1.44]) and higher-grade tumours (1.22 [1.08, 1.39]) compared with women without diabetes [164].
Norway					
HUNT ntnu.edu/hunt/	Kristian Hveem, Bjørn Olav Åsvold 1984 163,753	Nord-Trøndelag 54; Sør-Trøndelag 49	>18	Prospective population-based	3.3% of individuals with T2D had transient autoantibodies, were younger at diagnosis and showed beta cell dysfunction, suggesting a role of autoimmunity in disease heterogeneity [126]. Individuals with overweight and with high-risk genotypes (HLA, DR4/DR4) have a higher LADA risk; overweight also interacts with *TCF7L2* in T2D, highlighting the benefits of weight control in genetically susceptible people [108]. Low C-peptide and high GAD antibody levels predict LADA progression, while low C-peptide, younger age, high HbA_1c_ and obesity predict insulin dependence in T2D [165].
Scotland					
ET2DS usher.ed.ac.uk/molecular-epidemiology/our-studies/the-edinburgh-type-2-diabetes-study	Jackie Price 2006 1066	NA	60–75	Prospective population-based	IL-6-is associated with systemic inflammation and thought to accelerate age-related cognitive decline in T2D [76, 166]. Ultrasonography detects hepatic steatosis/MASLD; adding hyaluronic acid to FIB-4 enhances prediction of cirrhosis and hepatocellular carcinoma [167]. Biomarkers (cortisol, NT-proBNP, retinal traits) and metabolomics improve CVD risk prediction; retinal traits also aid retinopathy screening [168].
SDRN scottish-diabetes-research-network-t1-bioresource.ed.ac.uk/	Sarah Wild 2006 472,648	44.5	Mean age T1D 47, T2D 71	Population-based (health record database)	Individuals with no visible retinopathy in two consecutive screenings have a very low 2 year risk of disease progression, supporting screening for low-risk individuals every 2 years to reduce burden and optimise resources [31]. People with diabetes, especially T1D, face a markedly higher risk of severe COVID-19 outcomes, driven by factors such as older age, male sex, residential care, socioeconomic deprivation, comorbidities and smoking [135]. Analysis of over 500,000 people with T1D across 22 countries showed large variability, with children under 15 years achieving better control than adults, young adults lagging most and overall control still inadequate despite increased use of advanced diabetes technologies [169].
Sweden					
MPP malmo-kohorter.lu.se/malmo-cohorts	Olle Melander 1970s 33,000	33	46	Prospective population-based	Eleven gene variants (*TCF7L2*, *PPARG*, *FTO*, *KCNJ11*, *NOTCH2*, *WFS1*, *CDKAL1*, *IGF2BP2*, *SLC30A8*, *JAZF1* and *HHEX*) were found to be significantly associated with T2D risk, with eight of these variants linked to impaired beta cell function; adding genetic data modestly improves long-term prediction [109].
Malmö Diet and Cancer Study malmo-kohorter.lu.se/malmo-cohorts	Olle Melander 1991 28,000	60	57	Prospective population-based	Elevated fasting proneurotensin is linked to higher risk of T2D, CVD, breast cancer and mortality (especially in women), independent of other risk factors [170]. Metabolomic profiling identifies high-risk individuals (e.g. lean with obese metabolome) missed by BMI, improving T2D and mortality risk prediction [171].
SDPP ces.regionstockholm.se/projekt-och-uppdrag/sdpp/	Anton Lager Mid 1990s 34,486	56.3	35–56	Prospective population-based	High smokeless tobacco (snus) consumption, similar to smoking, raises T1D risk by impairing insulin secretion, showing snus is not a safe alternative to cigarettes [172]. Cluster-derived phenotypes effectively stratify T2D risk in diabetes-free adults and can inform targeted public health interventions. Six risk phenotypes were identified. Compared with the *LRHB* cluster, *VLR* and *LRLB* had low risk, *HRHBP*, *HRBF* and *HRIR* had high risk, with high-risk clusters predicting T2D more accurately than prediabetes over 20 years [173]. The most well-established behavioural and metabolic mediators explained only half of the socioeconomic health inequalities in the incidence of T2D [174].
ULSAM uu.se/en/research/ulsam	Lars Lind Early 1970s 2322	0	50	Prospective population-based	Obesity, IR and elevated systolic BP are major risk factors for T2D; some antihypertensive drugs may worsen risk via effects on insulin sensitivity. FHx increases risk through inherited IR [175]. High intact proinsulin is the strongest independent predictor of CHD morbidity/mortality and also predicts all-cause mortality better than insulin or immunoreactive insulin [176]. Elevated intact proinsulin and 32–33 split proinsulin strongly predict T2D, especially when acute insulin response is low, highlighting the value of monitoring proinsulin for early prevention [177].
VIP umu.se/brs/forskning/uttag-av-prover-och-data/diabnorr/	Olov Rolandsson 1985 ~14,000	51	40–60	Prospective population-based	Specialist validation (via DiabNorth register) improves diagnostic accuracy, reducing unspecified diabetes cases from 54.6% to 10.4% [178]. Increased dietary fibre intake and improved overall lifestyle behaviours are associated with a reduced risk of diabetes [91]. Screening detects diabetes ~4.6 years earlier, lowering risks of mortality, CVD, renal disease and retinopathy [24].

ADDITION-PRO, ADDITION-Progression; CHD, coronary heart disease; FHx, family history; FIB-4, fibrosis-4; GAD, glutamic acid decarboxylase; GDS, the German Diabetes Study; IR, insulin resistance; LADA, latent autoimmune diabetes in adults; LOFUS, Lolland-Falster Health Study; MARD, mild age-related diabetes; MOD, mild obesity-related diabetes; MPP, Malmö Preventive Project; NT-proBNP, N-terminal pro-B-type natriuretic peptide; PAEE, physical activity energy expenditure; SAID, severe autoimmune diabetes; SDPP, the Stockholm Diabetes Prevention Programme; SIDD, severe insulin-deficient diabetes; SIRD, severe insulin-resistant diabetes; T1D, type 1 diabetes mellitus; T2D, type 2 diabetes mellitus; ULSAM, Uppsala Longitudinal Study of Adult Men; VIP, Västerbotten Intervention Programme

**Table 2:** Non-European cohorts: characteristics and key lessons learned

Cohort name Link to cohort	Representative Year started Study population size	Female participants (%)	Age at recruitment (years)	Type of cohort	Important examples
Australia					
FDS uwa.edu.au/schools/research/fremantle-diabetes-study	Tim Davis Phase I 1993 1426 Phase II 2008 1732	NA	Mean 60	Prospective community-based	Four plasma biomarkers (ApoA4, CD5L, C1QB, IBP3) improve prediction of rapid kidney decline in T2D, aiding early detection and management [179].
					Since the 1990s, cardiovascular event rates in Australian individuals with T2D have improved, but all-cause mortality gaps persist, requiring focus on non-cardiovascular deaths [180].
					Four lung function patterns in T2D identified; severe decline linked to high HbA_1c_, smoking and obesity, increasing mortality and highlighting need for targeted interventions [181].
Brazil					
BDS clinicaltrials.gov/study/NCT04949152	Andrei C. Sposito 2016 1030 (as of 2021)	62	>30, mean 57	Prospective, single-centre, patient-based	Proteinuria increases diabetic macular oedema risk fourfold, independent of traditional risk factors [182].
					In T2D, carotid intimal thickness predicts coronary artery calcification better than intima–media thickness; carotid media thickness is not predictive [183].
					Low health literacy raises diabetic retinopathy risk in T2D, regardless of glycaemic management and other clinical factors [184].
Canada					
CLSA clsa-elcv.ca/	Parminder Raina 2010 51,338	52	45–85	Prospective, population-based	Diabetes raises depression risk in non-immigrants, while depression increases diabetes risk in both groups, more strongly in immigrants [61].
					Early T1D (<30 years) and early T2D (30–39 years) are linked to earlier menopause; T2D at ≥40 years is linked to later menopause; no link with gestational diabetes [185].
					T2D is associated with poorer executive function, slower reaction time and reduced short- and long-term memory [58].
China (Hong Kong)					
HKDR adf.org.hk/en/program/jadevision	Juliana C. N. Chan 1995 >30,000	55	>18	Register-based	The silent, progressive and complex nature of diabetes, with strong cognitive, psychosocial and behavioural influences, requires structured assessment to stratify risk, personalise care and identify unmet needs. Findings from the HKDR cohort and biobank highlight a distinct Asian diabetes phenotype characterised by young-onset disease driven more by genetics than autoimmunity, low BMI, high visceral fat and increased risk of stroke and kidney disease [186].
					Implementing this data-driven care model requires an improved practice environment, preferably day-centre settings rather than busy clinics, along with a nurse–clerk team to coordinate structured assessments (eye, foot, blood, urine) at presentation and every 2 years using a standardised checklist. In these settings, personalised data can support patient self-management and help clinicians identify unmet needs and prevent therapeutic inertia. Aggregated data can also inform administrators and payors about the model’s impact on reducing hospitalisations, morbidity and premature mortality [187].
Asia					
JADE Register adf.org.hk/en/program/jadevision	Juliana C. N. Chan 2007 >120,000	50	>18	Register-based	Integrating the HKDR protocol into the EMR system, along with establishing career paths for diabetes nurses and dedicated diabetes centres in 2000, expanded this academic quality-improvement initiative into a territory-wide risk assessment and management programme. It now serves over 600,000 people with diabetes in Hong Kong. This programme has contributed to a 50–70% reduction in acute and chronic complications, including mortality, and the resulting Hong Kong Diabetes Surveillance Database, containing glycaemic data from 4 million individuals, provides a valuable resource for trend analysis and real-world evidence [188].
					Digitalising the HKDR protocol into the web-based JADE platform in 2007, with risk stratification, decision support, event-probability estimates and data visualisation, has further scaled this data-driven care model across the region. Incorporating quality-of-life measures (EQ-5D) and hospitalisation costs in the JADE-HK cohort enabled development of the Chinese Diabetes Outcome Model, which accurately predicts lifetime risks of major complications and death to support precision management and cost-effectiveness analyses [132].
Ghana					
RODAM study rod-am.eu/	Charles O. Agyemang 2009 6250 Ghanaian individuals in five groups (1250 each) from rural/urban Ghana, Amsterdam, Berlin and London	NA	>25	Cross-sectional participant-based	Obesity and T2D increase from rural to urban Ghana, peaking among Ghanaian migrants in Europe, highlighting urgent public health needs [189].
RODAM-Pros study rod-am.eu/	2012 2165 participants plus 2098 European Dutch participants in a subsample	66	25–70	Prospective participant-based	Key DNA methylation sites, including *TXNIP* and the novel locus *TPM4*, are linked to T2D in sub-Saharan African individuals, offering targets for interventions [118].
					Groups of microbes that emerged or disappeared along the migration axis were associated with cardiometabolic outcomes, including higher BMI, higher HbA_1c_ and higher diastolic BP [190].
Greenland					
Greenland Population Health Survey sdu.dk/da/sif/rapporter/2011/inuit_health_in_transition	Marit Jørgensen 1993 2539	NA	>15	Cross-sectional population-based	Greenlandic Inuit-specific *ADCY3* c.2433–1G>A variant increases BMI, body fat, waist size and T2D risk, making *ADCY3* a potential obesity/diabetes target [77].
					Transition from active, traditional lifestyles to sedentary habits and dietary changes have increased obesity and T2D; public health efforts should promote traditional diets and activity [112].
					Greenlandic-specific c.1108G>T variant in *HNF1A* gene lowers insulin, raises glucose and increases T2D risk, explaining 2.5% of diabetes variance; high-impact variants cause ~18% of cases vs 1–3% in larger populations [97].
India					
CURES pubmed.ncbi.nlm.nih.gov/14710970/	R. M. Anjana 2001 26,000	50.6	≥20	Prospective community-based	Asian Indian individuals have high rates of diabetes/prediabetes with rapid progression; interventions should target modifiable risk factors [110].
					Many T2D cases are preventable via lifestyle changes, especially reducing central obesity and increasing activity [95].
					Diabetes raises cardiovascular mortality risk in Asian Indian individuals; early detection and management are essential [22].
Korea					
KoGES_Ansan and Ansung study NA	Ji Hee Yu 2001 10,030	52.6	40–69	Prospective population-based	Eight new loci identified (*GLIS3*, *PEPD*, *FITM2-R3HDML-HNF4A*, *KCNK16*, *MAEA*, *GCC1-PAX4*, *PSMD6* and *ZFAND3*) for T2D in East Asian individuals, revealing population-specific genetic factors [114].
					Impaired beta cell compensation for declining insulin sensitivity was identified as a key determinant of diabetes progression [191].
					Habitual sleep latency of 60 min or more was associated with significantly increased risks of all-cause and cancer-specific mortality over 18 years’ follow-up [192].
Singapore					
Diabetic Cohort - Singapore Population Health Studies blog.nus.edu.sg/sphs/population-studies/diabetic-cohort-dc/	Kavita Venkataraman 2004 14,033	49.2	>21, mean 59.7	Multicentre patient-based	rs10229583 (near *PAX4*): novel T2D locus in Chinese/East Asian individuals; linked to impaired beta cell function, high fasting glucose and earlier diagnosis [117].
					High baseline or worsening patterns increase risks of stroke, ESRD and mortality; Malay and Indian individuals are over-represented in high-risk groups [41].
					Annual transition probabilities of DKD progression were modelled across the KDIGO eGFR-defined risk stages. The majority (>50%) remained in the same stage annually, with the highest transition probabilities between stages G0 and G1 for both DKD progression and regression, which was the key driver of the DKD burden in Singapore [29].
MEC blog.nus.edu.sg/sphs/population-studies/multi-ethnic-cohort-phase-1-mec1/	Rob M. van Dam 2004 14,465	56	21–94	Closed cohort	Asian Indian individuals: higher IR and CRP, lower adiponectin; Chinese individuals: stronger BMI–metabolic links. Ethnicity-specific obesity management is needed [85].
					Three novel loci (*ADAM15*, *LINC02226*, *JUP*) + a known variant (*SLC4A1*) are linked to HbA_1c_; *SLC4A1* deletion may cause diabetes underdiagnosis via shorter erythrocyte lifespan [115].
					Malay and Indian individuals: ~2× higher T2D risk vs Chinese individuals due to adiposity, inflammation, IR, low beta cell function; Chinese individuals have lowest risk but lowest adiponectin levels, suggesting the presence of other protective factors [84].
USA					
ARIC Study aric.cscc.unc.edu/	Mary Rooney 1987 15,792	55	45–64	Prospective population-based	African American individuals, especially women, have a higher T2D incidence than White people; ~50% of excess risk in women is due to modifiable factors (adiposity), plus BP and lipid profile differences [83].
					Higher HbA_1c_ outperforms fasting glucose in predicting diabetes and CVD risk; elevated HbA_1c_ is linked to greater risks of diabetes, CHD, stroke and death [42].
					Single-sample confirmatory diagnosis of undiagnosed diabetes has high specificity and 15 year predictive value, linking confirmed cases to higher CVD, kidney disease and mortality risks [43].
HPFS hsph.harvard.edu/research/health-professionals/	Seyed Mohammad Mousavi 1986 51,529 US health professionals	Men only	40–75	Prospective population-based	Smoking increases diabetes risk and moderate alcohol consumption decreases diabetes risk, suggesting that lifestyle changes can impact prevention [92].
					Older adults: overweight may protect against mortality; unintentional weight loss may increase mortality risk [193].
NHS (including NHS, NHS II and HPFS) nurseshealthstudy.org/	Shilpa Bhupathiraju 1976 121,700 nurses	Female participants only	30–55	Prospective population-based	Most T2D cases can be preventable through a healthy lifestyle: weight control, healthy diet, physical activity, no smoking [90] (NHS).
					Lower saturated/trans fat and increased unsaturated fat consumption reduces CHD risk in men [194] (NHS).
					Increasing whole grain intake can significantly reduce the risk of T2D [195] (NHS, NHS II, HPFS).
					Replacing red meat with dairy, nuts and legumes reduces the risk of diabetes [89] (NHS, NHS II, HPFS).

BDS, Brazilian Diabetes Study; BP, blood pressure; CHD, coronary heart disease; CRP, C-reactive protein; CURES, Chennai Urban Rural Epidemiology Study; DKD, diabetic kidney disease; eGFR, estimated glomerular filtration rate; ESRD, end stage renal disease; FDS, Fremantle Diabetes Study; HPFS, Health Professionals Follow-up Study; IR, insulin resistance; KDIGO, Kidney Disease: Improving Global Outcomes; NHS, Nurses’ Health Study; RODAM-Pros, RODAM prospective; T1D, type 1 diabetes mellitus; T2D, type 2 diabetes mellitus

## Diagnosis and screening of (pre) type 2 diabetes

Many general population-based cohorts [4–14] were initially established to study the prevalence of prediabetes and type 2 diabetes and their associations with cardiovascular risk factors. Beyond measuring prevalence, these cohorts enabled systematic evaluation of individuals with undiagnosed prediabetes and undiagnosed type 2 diabetes and their cardiovascular risk profiles. For instance, data from the Cooperative Health Research in the Region of Augsburg (KORA) cohort (Germany) indicated that approximately 50% of individuals aged 55–74 years with diabetes were previously undiagnosed [15]. Similarly, the Intervention 1999 (Inter99) cohort (Denmark) and the Hoorn Studies (the Netherlands) reported that 65% of adults aged 30–60 years with diabetes were unaware of their condition [16, 17]. This knowledge helped drive improvements in diagnosis, screening and management of type 2 diabetes. A seminal advance in diabetes diagnosis was the introduction of HbA_1c_ as a diagnostic tool. The Hoorn Studies was among the first cohorts to establish HbA_1c_ as a determinant of retinopathy, which has informed the justification and determination of HbA_1c_ cut-off points for diabetes diagnosis [18]. The DETECT-2 study pooled data from nine cohorts across five countries on fasting plasma glucose, 2 h OGTT glucose and HbA_1c_, as well as retinopathy [19]. This study supported the 2009 International Expert Committee recommendation to use an HbA_1c_ threshold of 48 mmol/mol (6.5%) for diabetes diagnosis based on its consistent association with moderate or severe retinopathy [20] and escalating cardiovascular risk [21].

Early detection of type 2 diabetes has been shown to reduce comorbidities and all-cause mortality [22]. The Västerbotten Intervention Programme (Sweden) [23] targeting middle-aged residents demonstrated that individuals diagnosed with type 2 diabetes through screening fared better than those diagnosed clinically [24]. Individuals with screen-detected type 2 diabetes had lower rates of incident cardiovascular disease (CVD), nephropathy and retinopathy, and a twofold reduction in all-cause mortality [24]. In these individuals, intense treatment significantly improved cardiovascular outcomes over 5 years [25]. These findings emphasised the importance of early detection, which has led to many type 2 diabetes screening programmes. However, many cohort studies have selection bias, with under-representation of young people, workers and those with low socioeconomic status (SES) [26]. Given the rise of young-onset diabetes and importance of social determinants of type 2 diabetes, these historical cohorts may under-represent high-risk groups and be subject to lead-time bias, where early detection inflates apparent survival rate without necessarily improving outcomes. Finally, we need to recognise that universal screening for type 2 diabetes is not feasible, especially in low- and middle-income countries where the number of affected individuals is substantial. Use of validated risk scores such as the Indian Diabetes Risk Score, derived from the Chennai Urban Rural Epidemiology Study cohort (India), could help to make screening more precise and cost-effective [27]. Of note, multiple prospective cohorts have indicated that nearly 50% of people with diabetes in Asian cohorts were diagnosed only by 2 h plasma glucose during a 75 g OGTT [28]. Future recruitment strategies for new cohorts should prioritise diversity to ensure samples reflect young age groups, low SES and various ethnicities to avoid misrepresentation. Despite these limitations, insights from these historical cohort studies have shaped current diagnostic and clinical guidelines, with most professional organisations recommending targeted screening for individuals with risk factors, many of which have been elucidated through cohort studies. The text box on the diagnosis and screening of (pre) type 2 diabetes summarises key lessons learned from cohort studies.

**Figure:**
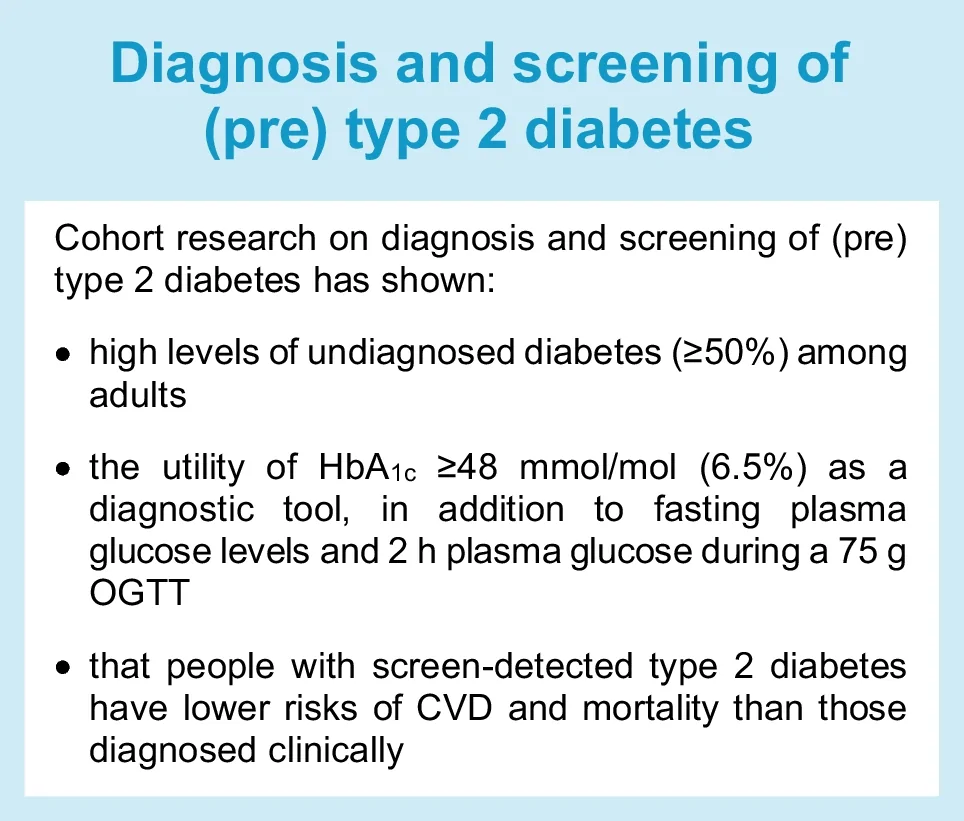

## Type 2 diabetes comorbidities

Cohort studies of people with established type 2 diabetes have advanced our understanding of comorbidities associated with type 2 diabetes. Microvascular complications of type 2 diabetes include nephropathy, retinopathy and neuropathy. Several cohorts showed high progression rates between nephropathy stages [29, 30], while individuals in another cohort with two consecutive screenings showing no retinopathy had a very low short-term risk of progressing to retinopathy within 2 years [31], informing targeted screening strategies. The German Diabetes Study linked early preclinical abnormalities with later development of peripheral neuropathy over a period of 10 years after type 2 diabetes diagnosis [32], suggesting that nerve dysfunction in well-controlled type 2 diabetes may reflect nerve status at diagnosis and ageing, more than ongoing glycaemic progression [33]. Other cohorts have implicated hyperglycaemia and other metabolic disturbances, especially central obesity, as drivers of polyneuropathy [34].

For macrovascular comorbidities, including coronary heart disease, stroke and peripheral arterial disease, cohort studies [34–46] such as the Framingham Heart Study have provided early and consistent insights into the elevated CVD risk in type 2 diabetes, with double the risk in men and almost triple the risk in women in comparison with those without type 2 diabetes [47]. The Hong Kong Diabetes Register (HKDR) was the first study to report a 1.5–2-fold higher risk of cardiovascular–renal complications and related death in Chinese people with young-onset type 2 diabetes diagnosed before age 40 years [48]. Likewise, a Swedish study using registry data from 2025 has indicated that type 2 diabetes before the age of 50 years confers a higher risk for CVD and heart failure than type 1 diabetes, underscoring higher vulnerability to CVD at younger ages in type 2 diabetes [49]. Large collaborations such as the Emerging Risk Factors Collaboration (Europe), which encompasses over 100 prospective cohorts, including several discussed in this review, further emphasise that the risk of premature death from all causes, including vascular disease, is higher among people with type 2 diabetes than among those without [35, 50]. Additionally, type 2 diabetes is associated with an accelerated decline in muscle mass [51] and strength [52], with sarcopenia emerging as an important comorbidity, particularly in older adults [53].

Across multiple cohorts, cancer emerges as a meaningful comorbidity in type 2 diabetes. The Emerging Risk Factors Collaboration (Europe) reported higher risks for several cancers (liver, pancreas, ovary, colorectum, bladder, lung and breast cancers) after adjusting for age, smoking status and BMI in those with type 2 diabetes compared with those without [50]. The Nurses’ Health Study (USA) and the Health Professionals Follow-up Study (USA) confirmed the elevated risks for liver, pancreas, colorectum, lung, oesophageal, thyroid and endometrial cancers, with the greatest risk occurring in the first 4–8 years after type 2 diabetes diagnosis, suggesting a possible mediating role of insulin resistance and hyperinsulinaemia [54]. People with type 2 diabetes had the highest risk for liver cancer, with a more than threefold increase compared with those without diabetes, probably attributable to coexisting metabolic dysfunction-associated steatotic liver disease (MASLD), previously known as non-alcoholic fatty liver disease (NAFLD). Cohorts such as the German Diabetes Study show a strong association between steatosis and insulin resistance in type 2 diabetes [36, 55]. Some cohorts also point to a potential causal role of hyperglycaemia in cancer risk, with attenuation observed with glucose-lowering drugs, statins and renin–angiotensin system inhibitors [56].

Type 2 diabetes has also been shown to have adverse effects on cognition and mental health, as well as other organ systems. The Atherosclerosis Risk in Communities (ARIC) study (USA) found that middle-aged adults with type 2 diabetes experienced a 19% greater decline in global cognitive function over two decades than their counterparts without diabetes [57]. Other cohorts have confirmed the reduction in cognitive function and its association with a decrease in white matter volume on MRI in type 2 diabetes [58, 59]. Depression is twice as prevalent among individuals with diabetes compared with the general population, affecting an estimated 20% of people with diabetes [60], with bidirectional associations uncovered between type 2 diabetes and depression [61]. A territory-wide cohort study involving 0.8 million people with type 2 diabetes and a matched cohort without type 2 diabetes found a higher hospitalisation rate for mental illness among people with type 2 diabetes, particularly among those aged under 40 years [62]. Additional comorbidities include a twofold increase in the risk of severe infection [63, 64], a higher incidence of fractures [65] and tendon rupture [66] and reduced pulmonary function [67] compared with individuals without type 2 diabetes. Importantly, cohorts from low- and middle-income countries face an enormous diabetes burden in settings with suboptimal detection and management resources [68, 69]. In summary, cohort studies have unveiled a broad, multisystem impact of type 2 diabetes across cardiovascular, oncological, cognitive, musculoskeletal, infectious and mental health domains. A key limitation of cohort studies is the potential oversight of additional comorbidities, as many cohort studies focus on specific outcomes (i.e. either cardiovascular or psychological). There is thus a need for cohort studies to adopt a broader spectrum of measurements, allowing for the discovery of unexpected associations. The text box on type 2 diabetes comorbidities summarises key lessons learned from cohort studies.

**Figure:**
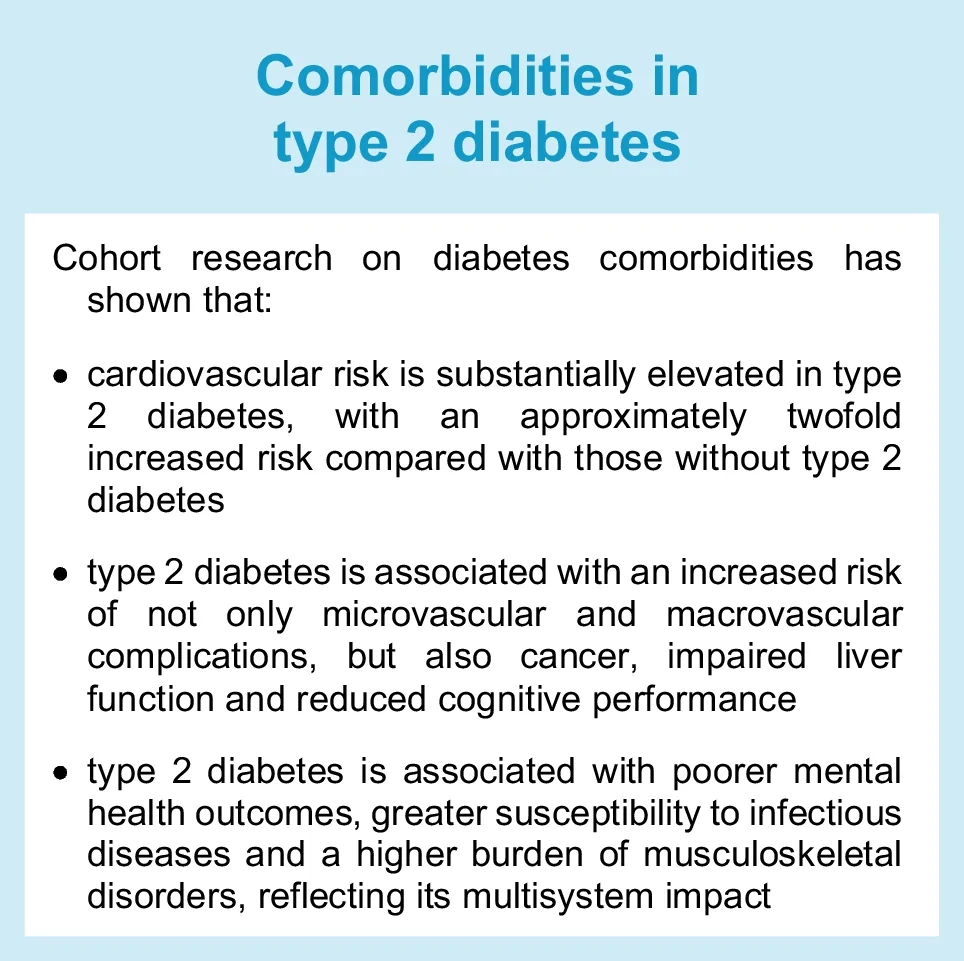

## Biological and socioeconomic risk factors for type 2 diabetes

Both early and later cohort studies have identified numerous risk factors for type 2 diabetes and its comorbidities. Core biological factors include impaired glucose regulation (prediabetes defined by elevated fasting plasma glucose, 2 h glucose during an OGTT or HbA_1c_) together with metabolic disturbances such as visceral adiposity and hypertension, as well as broader indicators of poor health, low birthweight and a family history of diabetes [15, 70, 71]. Weight trajectories, particularly overweight or obesity during early adulthood, are strongly associated with future type 2 diabetes [72, 73]. Similarly, systemic inflammation is associated with an elevated risk for type 2 diabetes [74] as well as its comorbidities [75]. For example, the Edinburgh Type 2 Diabetes Study (ET2DS) (Scotland) links IL-6 pathways to accelerated age-related cognitive decline in individuals with type 2 diabetes [76]. Finally, biomarker research spanning genomics, transcriptomics, metabolomics and proteomics, such as the FP7 IMI DIRECT project (Europe) [77], has deepened our mechanistic understanding and identified potential targets for personalised interventions, such as different profiles in post-load glucose predicting progression to diabetes [78]. However, translating these biomarkers into routine clinical practice remains complex and resource intensive, with only a few making it to implementation. One example is N-terminal pro-B-type natriuretic peptide [79], which has emerged as an important tool for diagnosis, risk stratification and therapeutic decision making in heart failure, the second most common form of CVD in people with type 2 diabetes.

The landscape of biological risk factors has expanded to encompass social determinants, revealing how racial, ethnic and socioeconomic disparities contribute to type 2 diabetes prevalence and incidence. For example, the Stockholm cohort (Sweden) found that economic status during early childhood, as indicated by the occupational positions held by fathers, was associated with a higher risk of type 2 diabetes in both male and female offspring [80]. In women, personal educational attainment was an important risk factor for type 2 diabetes [81]. In the Hong Kong Chinese sub-cohort of the Joint Asia Diabetes Evaluation (JADE) Register, years of education independently predicted cardiovascular and all-cause mortality [82], emphasising the importance of social determinants including general education in type 2 diabetes, with effects modulated by sex.

Ethnicity also modulates risk. The ARIC study (USA) demonstrated that African American women had higher incidence of type 2 diabetes than their white counterparts, with nearly half of the excess risk attributed to modifiable factors, such as adiposity [83]. Similarly, the Singapore Multi-Ethnic Cohort (MEC) (Singapore) found that Malay and Indian individuals had about twice the risk of developing type 2 diabetes than Chinese, associated with greater adiposity and inflammation [84]. By contrast, the associations between BMI and metabolic disturbances were stronger among Chinese individuals than Asian Indian individuals [85]. Indeed, the propensity of Asian Indian people to develop type 2 diabetes at lower levels of BMI has been termed the ‘South Asian phenotype’ and may relate to central adiposity and early loss of beta cell function [86]. This has even resulted in ethnic-specific cut-off points for obesity risk [87]. However, one limitation of this is that findings in Western cohorts have been relatively uncritically applied to non-Western populations, which for example leads to a heavy overestimation of type 2 diabetes based on HbA_1c_ in low-income countries, because HbA_1c_ is falsely high in iron deficiency [88]. There is thus a need for cohort studies to adopt a broader spectrum of socioeconomic factors and ethnicities to improve generalisability of results. The text box on biological and socioeconomic risk factors for type 2 diabetes summarises key lessons learned from cohort studies.

**Figure:**
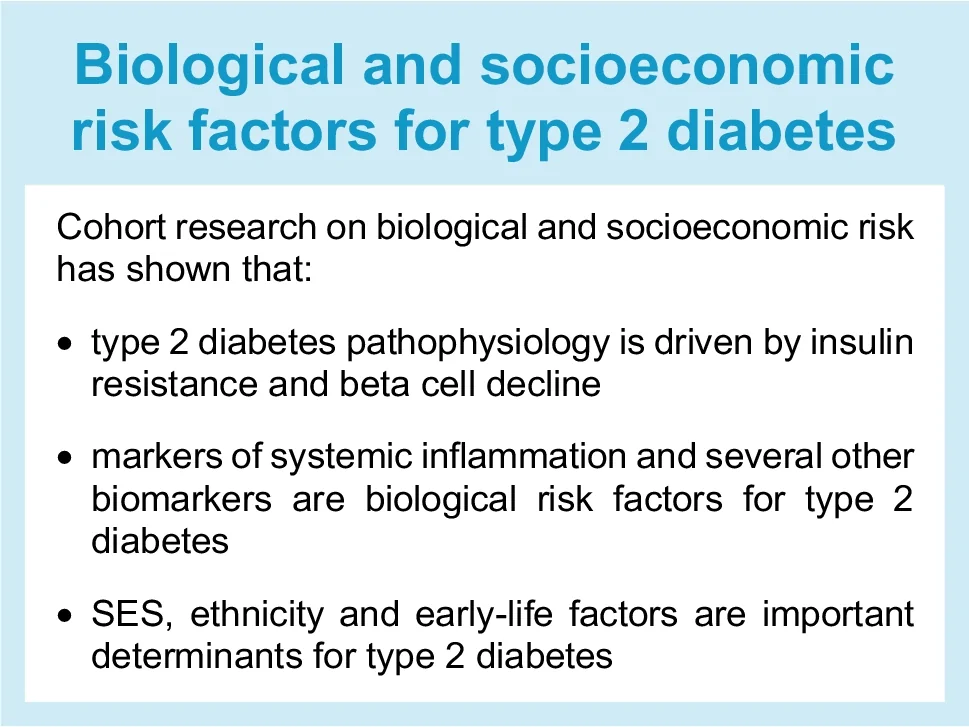

## Lifestyle and behavioural risk factors for type 2 diabetes

During the 1980s, researchers first reported the association of type 2 diabetes with smoking, followed by evidence that physical inactivity, alcohol intake and stress are independent risk factors for type 2 diabetes [15]. In the 1990s, studies revealed that healthier dietary choices were associated with reduced type 2 diabetes risk, including reduced red meat consumption [89], higher fibre intake [90–92], and lower glycaemic index and glycaemic load foods [93]. In the past decade, sleep has emerged as a new lifestyle factor. A recent meta-analysis found that both short (<6 h) and long (>8 h) sleep duration, insomnia, obstructive sleep apnoea (OSA) and irregular sleep timing were associated with a higher risk of type 2 diabetes, comparable to that observed with traditional risk factors [94]. In Asian Indian individuals, it has been estimated that more than 80% of incident diabetes could be prevented if five risk factors were favourably modified (obesity, physical inactivity, unfavourable diet risk score, hypertriglyceridemia and low HDL-cholesterol) [95].

These cohort data indicate the additive risks from multiple lifestyle factors. For example, the Västerbotten Intervention Programme (Sweden) showed a dose–response association between a lifestyle behaviour score and type 2 diabetes risk [91]. Analysis in the Nurses’ Health Study (USA) cohort suggested that the vast majority of cases of type 2 diabetes could be prevented by adoption of a healthy lifestyle [90]. They estimated that 91% (95 CI% 83, 95) of type 2 diabetes could be attributed to unhealthy habits and lifestyle behaviour, with genetics playing a less important role [90]. In the European Prospective Investigation into Cancer (Epic)-InterAct case–control study, with about 12,000 incident type 2 diabetes cases, there was no interaction between lifestyle and genetic factors [96]. Since many historical cohorts have excluded young people and non-Europeans, the significance of gene–environment–lifestyle interaction needs further exploration in these high-risk populations undergoing rapid societal, nutritional and lifestyle transitions. This is exemplified by the rising prevalence of type 2 diabetes in the Inuit population in Greenland as they shift from traditional active lifestyles to sedentary patterns, accompanied by a high-energy diet rich in processed foods [97].

Measurement of lifestyle factors remains challenging and susceptible to information bias. For example, diet assessment relies heavily on self-report and can be affected by under- or over-reporting of energy intake and dietary components, with confounding by education and SES. In summary, while observations from cohort studies generate important knowledge, well-designed randomised controlled trials (RCTs) are needed to establish causal relationships between these lifestyle factors and diabetes, with careful documentation of adherence to the lifestyle interventions. Looking ahead, advances in objective measurements (wearable devices for sleep and activity, continuous glucose monitoring, digital dietary tracking) and multi-omics analysis will increase the precision in measuring lifestyle factors. The text box on lifestyle and behavioural risk factors for type 2 diabetes summarises key lessons learned from cohort studies.

**Figure:**
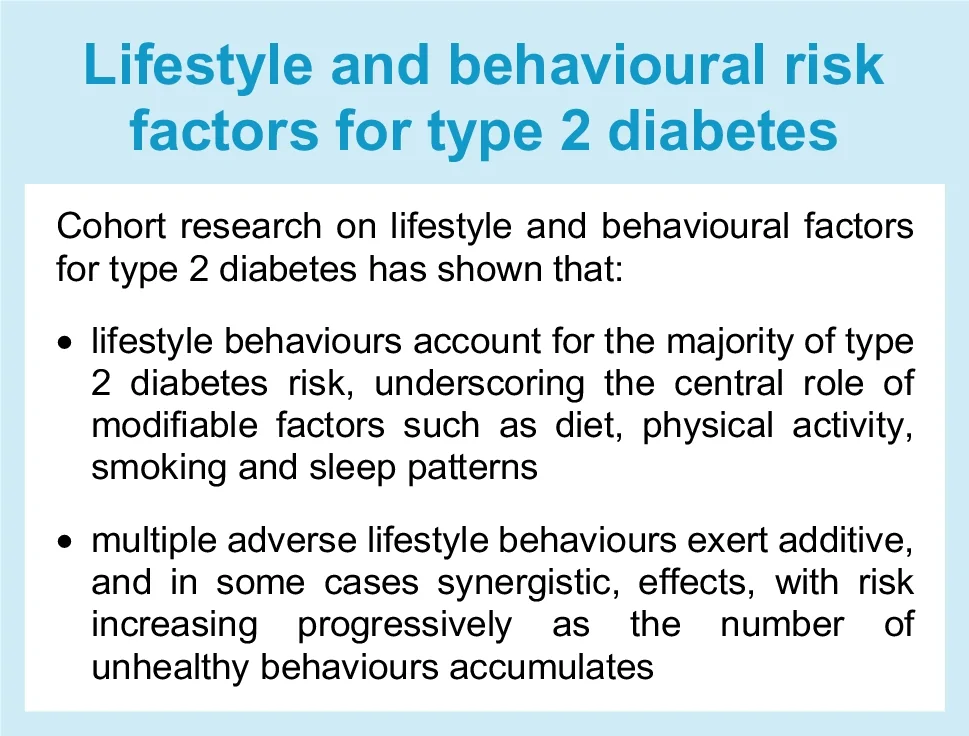

## Psychosocial risk factors for type 2 diabetes

In 2000, researchers from the Hoorn Studies (the Netherlands) highlighted a strong link between psychosocial stress and type 2 diabetes. In these participants, aged 50–75 years, those in the highest quintile of stressful life events (e.g. death of a partner, caring for chronically ill children, moving homes or job loss) were 1.5 times more likely to have (undetected) type 2 diabetes than those experiencing fewer stressors (lower four quintiles) [98]. In the Stockholm Diabetes Prevention Program cohort (Sweden), work-related stress as evidenced by low decision latitude and job strain was associated with a fourfold higher risk of type 2 diabetes, particularly among women [99]. These findings highlight the importance of individual psychological factors and the broader sociological context in precipitating and perpetuating risk of type 2 diabetes in predisposed individuals. In this light, the Canadian Longitudinal Study on Aging (CLSA) showed similar prevalence of diabetes in immigrants (16.4%) and non-immigrants (15.6%) [61]. However, type 2 diabetes was associated with a 27% higher risk of depression in non-immigrants, but not in immigrants [61], suggesting complex interactions between populations and health outcomes. The Helius Study (the Netherlands) also confirmed these nuances, showing that healthy and unhealthy lifestyle behaviours related to type 2 diabetes risk were differently clustered in Dutch, South Asian, African Surinamese, Ghanaian, Turkish and Moroccan people [100]. Taken together, inter-ethnic differences in cultures, perspectives and values may further modify these risk associations, especially in populations undergoing rapid transitions.

The unique strengths of cohort studies lie in their ability to examine long-term health outcomes, which could not be easily achieved in experimental research, which usually lasts for a few years only. For example, in the Hoorn Studies, individuals who experienced three or more stressful life events (i.e. death of spouse, disease, relationship problems) had 30–45% higher odds of mortality after 25 years compared with those with no stressful events [101]. Moreover, cohort studies can investigate factors that cannot ethically be manipulated in RCTs, such as differences in SES. For example, the Stockholm cohort revealed interactions between sex and SES for the association of psychosocial factors with diabetes [102]. Among men, the excess risk of type 2 diabetes in middle and low SES groups was partly explained by established risk factors, including BMI and lifestyle behaviours (accounting for 36–42%), whereas psychosocial factors contribute little to the association. In women, however, most of the SES-related differences in diabetes risk were explained by both established risk factors and psychosocial factors (81–100%). Together with the higher lifetime risk of complications in people with younger age of diagnosis, these cohort studies provide the rationale for designing experiments targeting specific groups to inform practice and policies [103]. However, observational research faces challenges related to confounding. A confounding variable is related to both the determinant and the outcome variable. This confounding variable causes a distortion of the true relationship between an exposure and a health outcome. Therefore, cohort studies need to be mindful about detailed ascertainment of variables that may act as confounders, and should be aware of unmeasured confounders. The text box on psychosocial risk factors for type 2 diabetes summarises key lessons learned from cohort studies.

**Figure:**
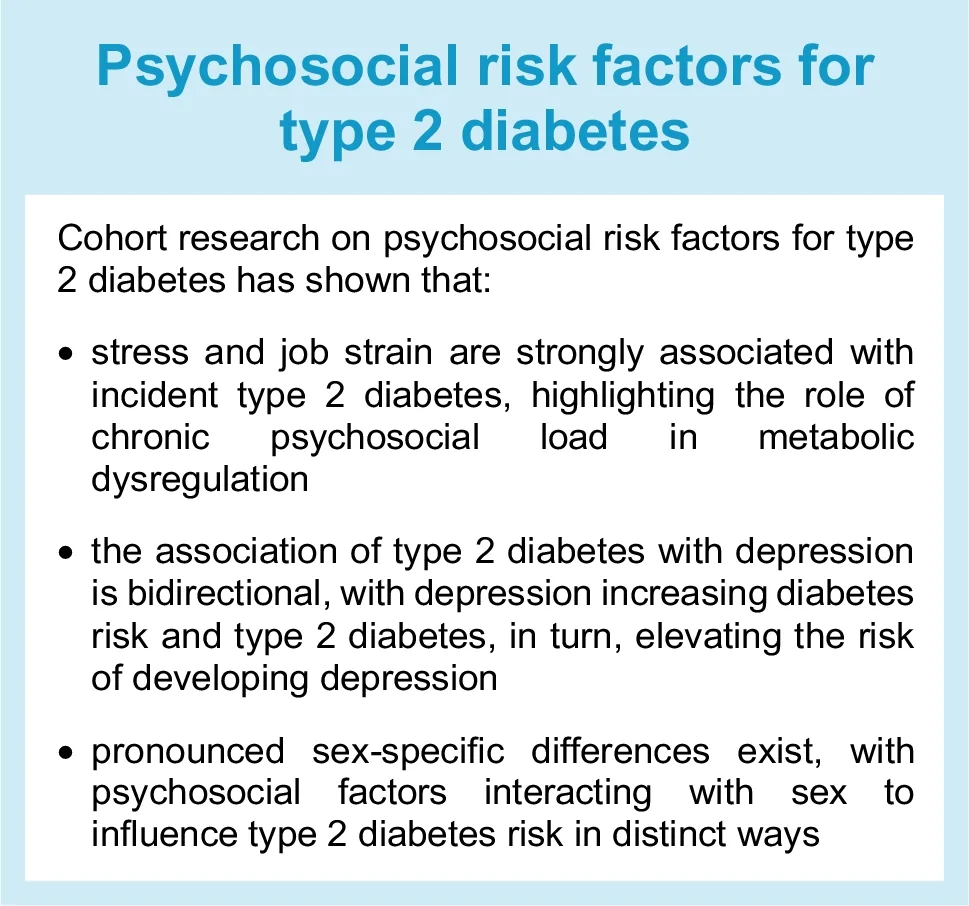

## Pathophysiology and genetics of type 2 diabetes

Cohort studies have greatly advanced the understanding of glucose homeostasis and the progression from normal glucose tolerance to impaired glucose regulation and diabetes. The European Group for the Study of Insulin Resistance: Relationship between insulin sensitivity and cardiovascular disease risk (EGIR-RISC) study (Europe) directly measured insulin sensitivity with euglycaemic–hyperinsulinaemic clamps and assessed pancreatic beta cell function using a five-point OGTT in more than 1000 European individuals [104]. Using mathematical modelling of beta cell function [107], the authors highlighted the importance of reduced beta cell glucose sensitivity (the slope of the glucose insulin secretion dose–response curve) for the loss of glucose tolerance and suggested that this occurs independently of changes in insulin resistance, challenging the dominant idea that the two variables are linked by a relationship of compensation [107]. In fact, the effects of insulin resistance and beta cell dysfunction on impaired glucose regulation were additive [105], which shows that time to glucose peak is influenced by insulin resistance, whereas the size of the glucose peak reflects both insulin sensitivity and secretion [106].

Given the central role of insulin sensitivity and pancreatic beta cell function in diabetes pathophysiology, extensive efforts have been made to uncover the genetic underpinnings. Several European studies [106, 108–112] have identified key genetic variants such as *TCF7L2*, *PPARG*, *CDKAL1*, *FTO* [106, 109] and *HNF1A* (c.1108G>T) [110], many of which are related to impaired beta cell function. A few, including *NAT2*, have been identified for insulin resistance, independent of BMI [113]. Interestingly, Asian cohorts, such as the Korean Genome and Epidemiology Study (KoGES) (Korea), Singapore Population Health Studies and the HKDR cohort and biobank, and the DC-Population Health Studies (USA) have identified new loci for type 2 diabetes, including *GLIS3*, *PEPD*, *KCNQ1*, *KCNK16*, *ADAM15*, *LINC02226*, *JUP* and *PAX4*, linked to insulin sensitivity and HbA_1c_ levels, highlighting East Asian-specific genetic factors [114–117]. Moreover, cohort studies have provided the first insights into the role of epigenetic factors in humans. The Research on Obesity and Diabetes among African Migrants (RODAM) study (the Netherlands) reported a risk association of DNA methylation in loci such as *TXNIP* and *TPM4* with type 2 diabetes unique in sub-Saharan Africans [118]. However, compared with other types of diabetes, such as type 1 diabetes or maturity-onset diabetes of the young, there are only a few studies that attempt to translate these genetic discoveries in clinical practice.

Finally, large consortia combining data from up to hundreds of cohorts, such as DIAGRAM and GENESIS, whole genome sequencing and more ancestrally diverse populations will help identify rarer variants involved in type 2 diabetes development and progression. However, there is a stark paucity of similar genetic data in non-European populations. These data are urgently needed for replication and discovery in our pursuit of precision prediction, prevention and care of diabetes in this global epidemic of type 2 diabetes [119]. Overall, these studies deepen our understanding of type 2 diabetes and related complications and support the move towards personalised care. In this context, the clinical utility and cost-effectiveness of incorporating these biomarkers in clinical practice needs to be systematically evaluated in experimental and implementation studies. Furthermore, the process of translating this evidence to practice is complex and resource intensive. The text box on the pathophysiology and genetics of type 2 diabetes summarises key lessons learned from cohort studies.

**Figure:**
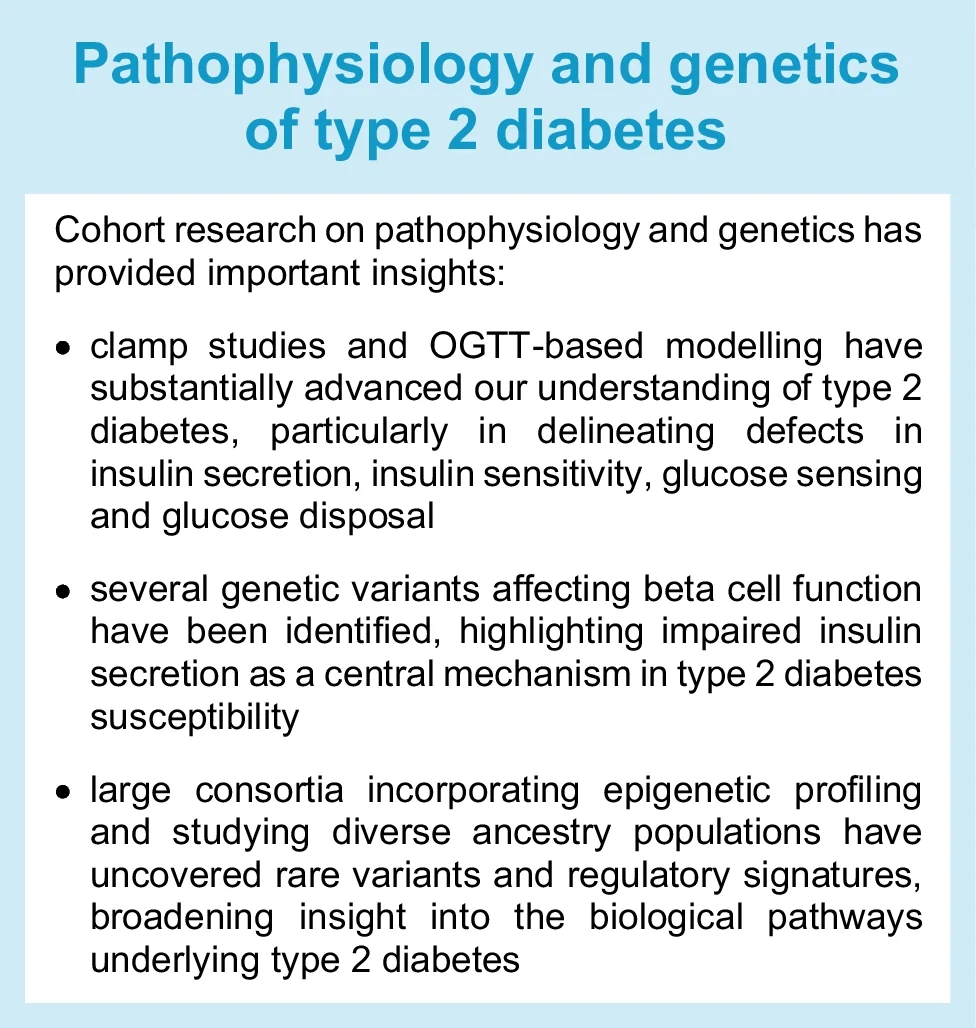

## Emerging research and future directions

Over the past 50 years, cohort research advanced diagnosis and screening of type 2 diabetes, identification and prevention of comorbidities, and knowledge on risk factors and pathophysiology. New developments in information and communication technology and statistical methods can enable complex analysis of large datasets gathered over long periods. This progress will facilitate creation of complex constructs related to an individual’s total environment, often referred to as the ‘exposome’, and its associations with outcomes. The exposome represents the (measurable) totality of environmental, or nongenetic, drivers of health and disease, and can be subdivided as the external and internal exposomes. The external exposome includes the built environment, social environment, physical–chemical environment and lifestyle/food environment [120]. The internal exposome encompasses measurements at the epigenetic, transcriptomic, proteomic, microbiomic or metabolomic levels. The impacts of these internal and external exposomes are in part mediated through cognitive–psychosocial–behavioural processes with heterogenous expression in terms of biomarkers, traits, phenotypes and outcomes. Measurements of these multi-dimensional data enable researchers to analyse how these exposures might imprint upon the host’s biology and how the host might respond to these environmental insults, resulting in diverse biological responses, which in turn might be influenced by their genetic make-up. In a narrative review, Beulens et al. [120] highlighted the importance of the exposome as a risk factor for type 2 diabetes. However, most of these associations were cross-sectional in nature, which highlights a need for research into the exposome using comprehensive cohort measurements. According to Hill’s criteria on causality, prospective and experimental cohorts were more informative than cross-sectional analysis in the evidence cascade, aimed at changing dogma, practice and policies [121].

A second promising direction for cohort research concerns the development of precision health approaches, encompassing precision prevention and treatment, tailored to the individual characteristics of each person. One example is the cluster analysis by Ahlqvist et al, which identified five subtypes of type 2 diabetes in the ANDIS cohort (Sweden) [122]. These subtypes have been validated against gold-standard measures of insulin sensitivity and beta cell function in the German Diabetes Study [123], with replication in other populations [124]. These clusters were associated with different genetic profiles, comorbidities and responses to treatment. These included early insulin treatment in individuals with insulin-deficient diabetes and a high risk for nephropathy subtype. However, depending on the variables used for cluster analysis, other studies such as the Danish Centre for Strategic Research in Type 2 Diabetes (DD2) (Denmark) have proposed alternative type 2 diabetes subphenotypes [125]. In the Trøndelag Health Study (HUNT) (Norway), serum collected prior to type 2 diabetes onset revealed that 3% of people with type 2 diabetes had previous, but transient, evidence of autoantibodies, suggesting a role of autoimmunity in type 2 diabetes disease heterogeneity [126]. However, the components of these clusters vary depending on types of available data. On the other hand, Dennis et al suggested that precision medicine in type 2 diabetes is likely to have most clinical utility if it is based on an approach of using specific phenotypic measures to predict specific outcomes, rather than assigning individuals to subgroups [127].

Another example of precision health relates to HbA_1c_ reduction in response to glucagon-like peptide 1 receptor agonist (GLP-1RA). Cohort analysis revealed that carriers of the *GLP1R* gene had a greater reduction in HbA_1c_ than non-carriers [128]. Given the growing number of glucose-lowering drugs, analysis of prospective cohorts with detailed phenotypes, treatment and outcomes is an important resource for analysis related to risk stratification and disease classification for targeted therapies or interventions. New technologies are now generating multi-omics data (e.g. metabolomics, proteomics, epigenetics) from cohort studies which are rapidly shaping biomarker discovery and precision health [129]. However, to date only a few biomarkers have made it into practice, due to high costs or barriers to clinical applicability.

A third future direction for cohorts involves the use of real-world data to evaluate risks and benefits of type 2 diabetes interventions. In 1973, Weddell first explained the need to establish disease registers to identify patterns and trends for planning of prevention and treatment services [130]. With increasing use of electronic medical record (EMR) systems, which often capture medication, procedure, laboratory, hospitalisation and death data, establishing a register with well-defined variables and exposures based on prior knowledge will provide effectiveness and cost-effectiveness data to complement the efficacy and safety data of RCTs. Typical examples are the UK Prospective Diabetes Study (UKPDS) Outcome Model and the Chinese Diabetes Outcome Model based on the HKDR, with both cohorts having more than three decades of follow-up [131, 132].

In most RCTs, young people, geriatric and frail people, and minor ethnic groups are often under-represented along with exclusion of various drugs and people with comorbidities. With increasing use of novel drugs, register-based cohorts can provide essential real-world data regarding weight-reducing and metabolic effects of drugs such as GLP-1RA and their adverse effects or complications [133]. Registers can also reveal novel insights, for example, in the DIAbetes MANagement and Treatment (DIAMANT) cohort (the Netherlands), people with type 2 diabetes had a 1.3 times higher risk of developing colorectal cancer than those without type 2 diabetes [134]. During the COVID-19 pandemic, the Scottish Diabetes Research Network (SDRN) cohort (Scotland) reported the risk associations of type 2 diabetes with severe COVID-19 outcomes, further increased by older age, male sex, residential care, higher deprivation, comorbid conditions and smoking [135]. Here it is important to distinguish between purpose-built registers and unstructured registers. Unless the registers have a built-in protocol for systematic data collection, most consist of non-standardised measurements with many missing data. Often these registers are merged with probabilistic matching to provide real-word evidence to complement RCT data, although not without bias. In this context, practising physicians who have integrated knowledge of human biology, clinical medicine and treatments should familiarise themselves with the principles of epidemiology and help design protocols for establishing registers. To ensure fidelity, improvement of the practice environment and training of staff during data collection will help close gaps in prevention, care and professional knowledge [136].

Finally, we are witnessing the emergence of new forms of cohorts, which combine observational and interventional designs. For example, the Inter99 cohort (Denmark) and the Anglo–Danish–Dutch Study of Intensive Treatment In People with Screen-Detected Diabetes in Primary Care (ADDITION-Europe) cohort [137–141] reported risk profiles of participants and results of interventions in subgroups participating in a lifestyle intervention. This type of research is needed to assess the long-term or legacy effects of interventions given that most RCTs last for only 2–5 years. Other examples include the web-based JADE Platform which incorporated the HKDR protocol for establishing cohorts in Asia, with some registrants participating in interventions with long-term data on their benefits [136]. Another important development in cohort research is the emergence of Developmental Origins of Health and Disease (DOHaD) cohorts. DOHaD cohorts are longitudinal research studies that follow individuals, often from preconception, pregnancy or birth, throughout their life course to examine how early-life environmental, nutritional and social exposures shape long-term health outcomes, including type 2 diabetes.

Despite the development of new cohorts or expansion of existing cohorts, including multi-omics analysis and embedded intervention(s), there is an urgent need to establish cohorts especially in low-income and middle-income countries and with more diverse and representative populations. New cohorts need a clear aim and hypothesis and to obtain ethical approval. They should define a target population with careful design and well-defined recruitment strategies to avoid selection bias. These protocols should consider objective and validated measurement of exposures, confounders and outcomes. Well-designed and implemented observational research requires substantial resources, including time, funding and personnel. Most importantly, these research programmes should be guided by well-informed investigators committed to continuous learning and using research to change practice. Sharing of experiences by investigators of existing cohorts with new researchers may expedite the set-up, management and analysis of new cohorts to advance knowledge and practice.

In the past decade, with growing interest in using real-world data for research, regulations on data governance based on the FAIR (Findable, Accessible, Interoperable and Reusable) principles as well as ethics regarding data collection and sharing have been reformulated. With increasing popularity of using real-word evidence for research or practice, we anticipate that more cohort collaborations with data sharing, individual or aggregate, will generate complex datasets for knowledge discovery and application. The text box summarises our call to action for the future of cohort research.

**Figure:**
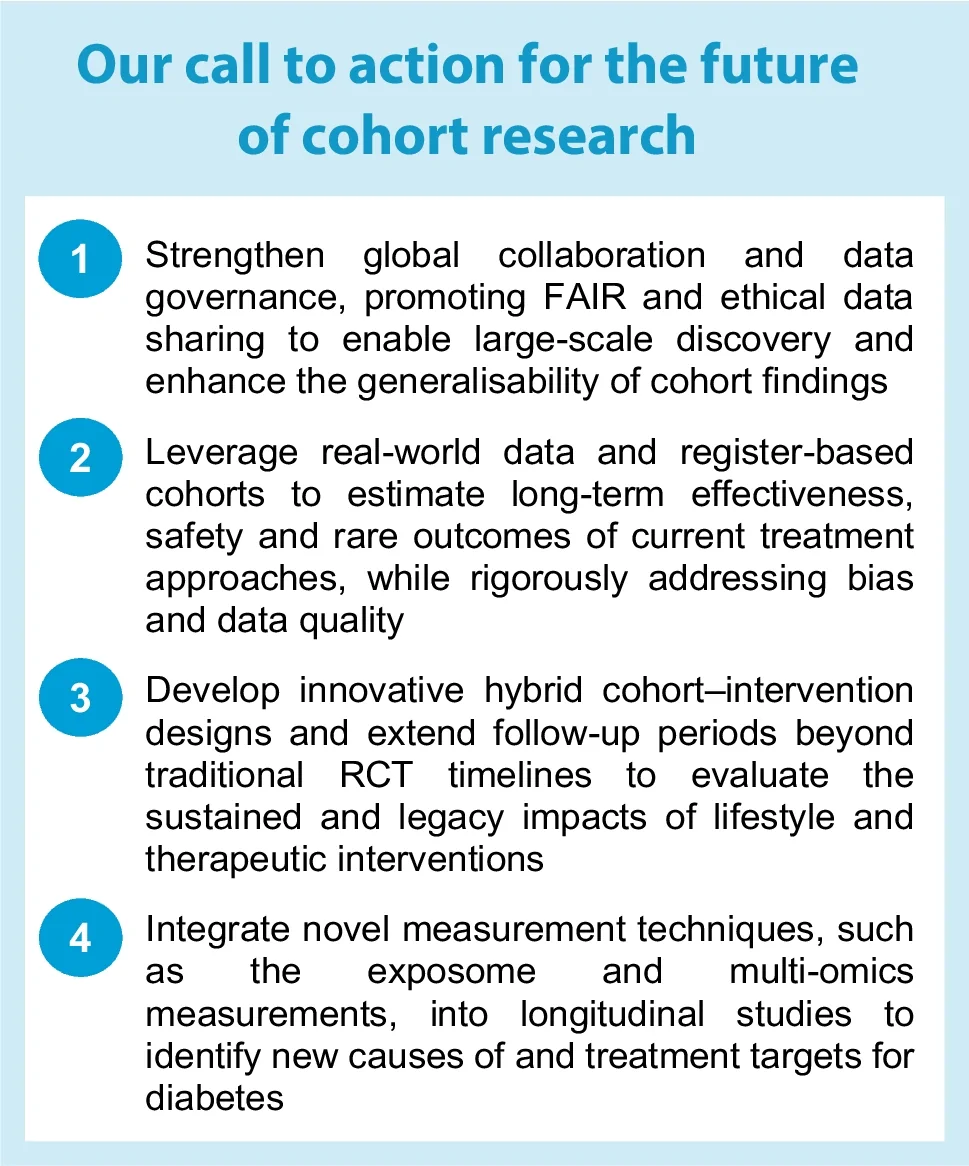

In this review, we aimed to be as inclusive as possible in selecting cohorts and summarising key findings. This is, however, not a systematic review and we acknowledge that many cohorts and findings that have also contributed to the field might have been missed. The lack of inclusion of these cohorts and findings does not diminish their important contributions to diabetes research. Notwithstanding these limitations, we have demonstrated the importance of well-designed cohort studies by summarising results from the past 50 years around several themes. We also discussed the strengths and weaknesses of observational research in comparison with other research designs. This wealth of data has contributed to the evolution of diagnosis, classification, prevention and treatment of type 2 diabetes. We conclude that cohort research has significantly advanced our understanding of type 2 diabetes, aided guideline development and complemented experimental work, such as human RCTs and animal studies. These observational and experimental approaches are essential and complementary in our pursuit to provide a more comprehensive understanding of the development and progression of type 2 diabetes and to change dogma, practice and policies for better outcomes.

## Abbreviations

ADDITION-Europe: Anglo–Danish–Dutch Study of Intensive Treatment In People with Screen-Detected Diabetes in Primary Care
ARIC: Atherosclerosis Risk In Communities
CLSA: Canadian Longitudinal Study on Aging
CVD: Cardiovascular disease
DD2: Danish Centre for Strategic Research in Type 2 Diabetes
DIAMANT: DIAbetes MANagement and Treatment
DOHaD: Developmental Origins of Health and Disease
EGIR-RISC: European Group for the Study of Insulin Resistance: Relationship between insulin sensitivity and cardiovascular disease risk
EMR: Electronic medical record
ET2DS: Edinburgh Type 2 Diabetes Study
FAIR: Findable, Accessible, Interoperable and Reusable
GLP-1RA: Glucagon-like peptide 1 receptor agonist
HKDR: Hong Kong Diabetes Register
HUNT: Trøndelag Health Study
Inter99: Intervention 1999
JADE: Joint Asia Diabetes Evaluation
KoGES: Korean Genome and Epidemiology Study
KORA: Cooperative Health Research in the Region of Augsburg
MASLD: Metabolic dysfunction-associated steatotic liver disease
MEC: Singapore Multi-Ethnic Cohort
RCT: Randomised controlled trial
RODAM: Research on Obesity and Diabetes among African Migrants
SDRN: Scottish Diabetes Research Network
SES: Socioeconomic status
